# Letter by Neugebauer et al. regarding article “Hypothermia after decompressive hemicraniectomy in treatment of malignant middle cerebral artery stroke: comment on the randomized clinical trial”

**DOI:** 10.1186/s13054-019-2600-9

**Published:** 2019-09-17

**Authors:** Hermann Neugebauer, Hauke Schneider, Rainer Kollmar

**Affiliations:** 1 Department of Neurology, University of Würzburg, Josef-Schneider-Str. 11, 97080 Würzburg, Germany; 2 Department of Neurology, University Hospital Augsburg, Augsburg, Germany; 3 Department of Neurology and Neurointensive Care, Klinikum Darmstadt, Darmstadt, Germany

**Keywords:** Stroke, Hypothermia, Hemicraniectomy

*To the Editor,*

Therapeutic hypothermia was thought to be one of the most promising treatment strategies for acute ischemic stroke because of beneficial multimodal effects in animal studies and successful clinical trials in cardiac arrest. We had to report negative trial results on hypothermia following hemicraniectomy in space-occupying stroke [1].

Engrand et al. raised concerns about the blood gas management and the timing of hypothermia in our trial [2]. We want to address these concerns as follows:

First, most knowledge on blood gas management derive from studies on transient global anoxia after cardiac arrest and cardiac surgery. In contrast, space-occupying stroke arising from permanent focal ischemia is different regarding the underlying pathophysiology and histopathology [3]. There is only one clinical study on the effects of blood gas management in patients with space-occupying stroke reporting no outcome data but particularly none after hemicraniectomy, the most effective intracranial pressure-lowering therapy [4]. In patients without hemicraniectomy, pH-stat management was beneficial in the first 2 days after stroke and associated with a critical increase in intracranial pressure thereafter. Increased intracranial pressure was immediately responsive to a change in ventilation strategy.

Second, although we used temperature-corrected pH-stat management, it is common sense that ventilation and blood gas management is tailored to ICP values in patients with raised intracranial pressure.

Third, hypothermia is characterized by its multimodal pathophysiological effects on the ischemic cascade lasting far beyond the first hours of stroke. Besides neuroprotective effects early after ischemia, hypothermia prevents brain-edema by attenuating inflammatory processes and stabilization of the blood-brain barrier independent of blood gases [5].

Therefore, it is speculative to state that pH-stat management was the major reason for severe adverse events in our trial. If the concerns were applicable to our situation at all, the main issue of brain-edema was met by hemicraniectomy and tailored ventilation. Mortality rates and central nervous system adverse events including hemorrhagic transformation did not differ significantly between treatment groups. We think that the overall increased rate of severe adverse events is related to prolonged sedation, rebound edema during rewarming, or other side effects of hypothermia such as detrimental cardiovascular events. It is quite possible that hypothermia is more effective the earlier it is applied. However, this is a practical issue as moderate hypothermia can hardly be applied to awake stroke patients, space-occupying brain-edema cannot be predicted with adequate sensitivity to allow (ultra-)early hemicraniectomy, and surgery under hypothermic conditions may be complicated by bleeding.

## Data Availability

N.A.
